# ID4 Is Required for Normal Ependymal Cell Development

**DOI:** 10.3389/fnins.2021.668243

**Published:** 2021-04-22

**Authors:** Brenda Rocamonde, Vicente Herranz-Pérez, Jose Manuel Garcia-Verdugo, Emmanuelle Huillard

**Affiliations:** ^1^INSERM, CNRS, APHP, Institut du Cerveau – Paris Brain Institute (ICM), Sorbonne Université, Paris, France; ^2^Laboratory of Comparative Neurobiology, CIBERNED, Institute Cavanilles, University of Valencia, Valencia, Spain; ^3^Predepartamental Unit of Medicine, Faculty of Health Sciences, Jaume I University, Castellón de la Plana, Spain

**Keywords:** ID4, ependymal cell, development, brain, transcription factor

## Abstract

Ependymal cells are radial glia-derived multiciliated cells lining the lateral ventricles of the brain and spinal cord. Correct development and coordinated cilia beating is essential for proper cerebrospinal fluid (CSF) flow and neurogenesis modulation. Dysfunctions of ependymal cells were associated with transcription factor deregulation. Here we provide evidence that the transcriptional regulator ID4 is involved in ependymal cell development and maturation. We observed that *Id4*-deficient mice display altered ventricular cell cytoarchitecture, decreased ependymal cell number and enlarged ventricles. In addition, absence of ID4 during embryonic development resulted in decreased ependymal cell number and delayed maturation. Our findings open the way for a potential role of ID4 in ependymal cell development and motor cilia function.

## Introduction

Ependymal cells are multiciliated epithelial cells organized in a monolayer lining the lateral ventricles (LV) (Doetsch et al., 1997). A subpopulation of radial glia-derived B1 astrocytes present an apical membrane extending a primary cilia that contacts the ventricle (Doetsch et al., 1999; Merkle et al., 2004). Monociliated B1 astrocytes and multiciliated ependymal cells are organized within the neurogenic regions of the ventricle wall forming unique pinwheel structures (Mirzadeh et al., 2008).

Ependymal cells are derived from radial glia during embryogenesis between embryonic day 14 (E14) and E16, while maturation occurs later during the first postnatal week. Ependymal cells are born as non-motile monociliated epithelial cells (9 + 0 microtubule doublets) and their maturation as motile multiciliated cells (9 + 2 microtubule doublets) occurs during postnatal day 0 (P0) and P10 (Spassky et al., 2005). It was reported that rotational and translational orientation of basal bodies (BB) are determinant factors of planar cell polarity (PCP) and correlate with cerebrospinal fluid (CSF) flow direction. The coordinated ependymal cell beating is responsible for the correct CSF flow through the ventricular system. CSF flow disruption due to ependymal cell malfunction can lead to hydrocephaly (Taulman et al., 2001) and could impact neuroblast migration toward the olfactory bulb (OB) (Sawamoto et al., 2006).

Factors controlling differentiation and maturation of ependymal cells are not well characterized. It is known that the forkhead transcription factor FOXJ1 is necessary for ependymal cell differentiation from radial glial cells and ciliogenesis (Jacquet et al., 2009). The homeobox factor SIX3 and the transcription factor nuclear factor IX (NFIX) are also involved in ependymal cell development and maturation (Lavado and Oliver, 2011; Vidovic et al., 2018). More recently, it was demonstrated that Geminin and its antagonist GemC1, which are regulators of DNA replication, can determinate the proportion of ependymal cells and neural stem cells (Ortiz-Álvarez et al., 2019).

Inhibitor of DNA-binding 4 protein (ID4) is a helix-loop-helix (HLH) protein, acting as a binding partner and modulator of bHLH transcription factors. During embryonic development, ID4 plays an important role in the development of the central nervous system, regulating neural stem cell (NSC) proliferation and differentiation. ID4-deficient mice present premature differentiation and compromised cell cycle transition of early progenitor cells resulting in smaller brain (Yun et al., 2004; Bedford et al., 2005). However, the role of ID4 in ependymal cell development and maturation from radial glia cells has not yet been addressed. Here we show that ID4 is necessary for correct development of the LV epithelium and for correct ependymal cell maturation. Absence of ID4 during crucial stages of neural fate decision leads to defective ependymal cell development, disrupted PCP and hydrocephalus. Our data suggest for the first time a role for ID4 in ependyma development and function.

## Experimental Procedures

### Animals

Mice were housed, bred, and treated in an authorized facility (agreement number A751319). All experimental procedures involving mice have been approved by the French Ministry of Research and Higher Education (project authorization number 3572-201601 0817294743 v5). C57BL/6J (Charles River laboratories) and *Id4−/−* (Yun et al., 2004) mice were used at 2–3 months of age. *Glast:CreERT2* (Mori et al., 2006) mice bred to *Id4fl* mice (Best et al., 2014) to obtain *Glast:CreERT2;Id4fl* mice. To induce Id4 deletion, a solution of 60 mg/kg of tamoxifen and 20 mg/kg of progesterone diluted in corn oil was administered by oral gavage to pregnant females harvesting embryos at embryonic day 15 (E15). Pups were then obtained at P0 or P10 for wholemount dissection.

### Wholemount Dissection and Immunolabeling

Animals were sacrificed by cervical dislocation. Then, brain was dissected and the whole ventricular-subventricular zone (V-SVZ) was microdissected as described in Mirzadeh et al. (2010). Fresh tissue was either fixed with 4% paraformaldehyde (PFA; Electron Microscopy Sciences, EMS) and incubated with anti- ZO-1 (1:200, Thermo Fisher Scientific ref. 402200); acetylated tubulin (6-11B, 1:200, Sigma Aldrich ref. T6793) primary antibodies; or fixed with cold 70% ethanol for 10 min and incubated with the anti-gamma tubulin (GTU88, 1:200, Abcam ref. ab11361) primary antibody. Samples were incubated with secondary antibodies Alexa Fluor^TM^ 488 and 596 (1:1000, Life Science Technologies). Then wholemount sections were microdissected and mounted with fluoromount (Sigma, ref. F4680). Planar cell polarity was determined by the altered orientation of the BB with respect of the cell wall as described in Mirzadeh et al. (2010).

### Immunofluorescence

Animals were anesthetized with 1 g/Kg sodium pentobarbital (EUTHASOL) and intracardially perfused with 4% PFA in NaCl 0.9% solution. Brains were dissected and post-fixed in the same solution for 24 h. Fifty micron coronal sections were obtained using a vibratome (Microm). Floating sections were permeabilized with 0.01 M phosphate buffer saline (PBS) containing 0.1% Triton X-100 for 5 min, blocked for 1 h with 10% Normal Goat Serum (Eurobio Ingen, cat. CAECHV00-0U) in PBS–Triton 0.1% (blocking buffer) at room temperature RT and incubated overnight at 4°C with rabbit anti-ID4 (1:1000, Biocheck ref. BCH-9/82-12) and mouse anti-FOXJ1 (1:500, eBiosc 14-9965-82) antibodies diluted in blocking buffer. Then samples were incubated with anti-mouse Alexa Fluor^TM^ 488 and anti-rabbit Alexa Fluor^TM^ 596 secondary antibodies (1:1000, Life Science Technologies) diluted in blocking buffer. Finally, sections were incubated in DAPI solution for nuclear staining (Invitrogen, cat D3571) and mounted on glass-slides with Fluoromount (Sigma, cat. F4680).

### Electron Microscopy and Immunogold Staining

For scanning electron microscopy, whole-mount preparations of the lateral wall of lateral ventricles of four animals per group were dissected and fixed with 2% PFA + 2.5% glutaraldehyde (EMS) in 0.1 M phosphate buffer (PB) and post-fixed with 1% osmium tetroxide (EMS) in PB for 2 h, rinsed with deionized water, and dehydrated first in ethanol then with CO_2_ by critical point drying method. The samples were coated with gold/palladium alloy by sputter coating. The surface of the lateral wall was studied under a Hitachi S-4800 scanning electron microscope using Quantax 400 software (Bruker Corporation) for image acquisition.

For pre-embedding immunogold staining, mice were perfused with 4% PFA in 0.1 M PB. Brains were postfixed in the same fixative solution overnight at 4°C and sectioned into 50 μm transversal sections using a vibratome. Pre-embedding immunogold staining with rabbit anti-ID4 antibody (1:500; Biocheck) were carried out as previously described (Sirerol-Piquer et al., 2012). Sections were contrasted with 1% osmium tetroxide, 7% glucose in 0.1 M PB and embedded in Durcupan epoxy resin. Subsequently, 1.5 μm semithin sections were prepared, lightly stained with 1% toluidine blue and selected at the light microscope level. Selected levels were cut into 60–80 nm ultrathin sections. These sections were placed on Formvar-coated single-slot copper grids (EMS) stained with lead citrate and examined at 80 kV on a FEI Tecnai G^2^ Spirit (FEI Company) transmission electron microscope equipped with a Morada CCD digital camera (Olympus).

### Image Acquisition and Analysis

Fluorescent images were obtained using a Zeiss ApoTome 2 Microscope or Olympus Confocal microscope FV1000. Images were analyzed using ImageJ software.

### Statistical Analysis

Statistical analysis was performed using GraphPad Prism 6. Unless otherwise indicated in the figure legends, non-parametric Mann–Whitney test was used to compare experimental and control groups. Values are expressed as mean ± standard deviation.

## Results

### ID4 Is Expressed in Ependymal Cells From the LV

**FIGURE 1:**
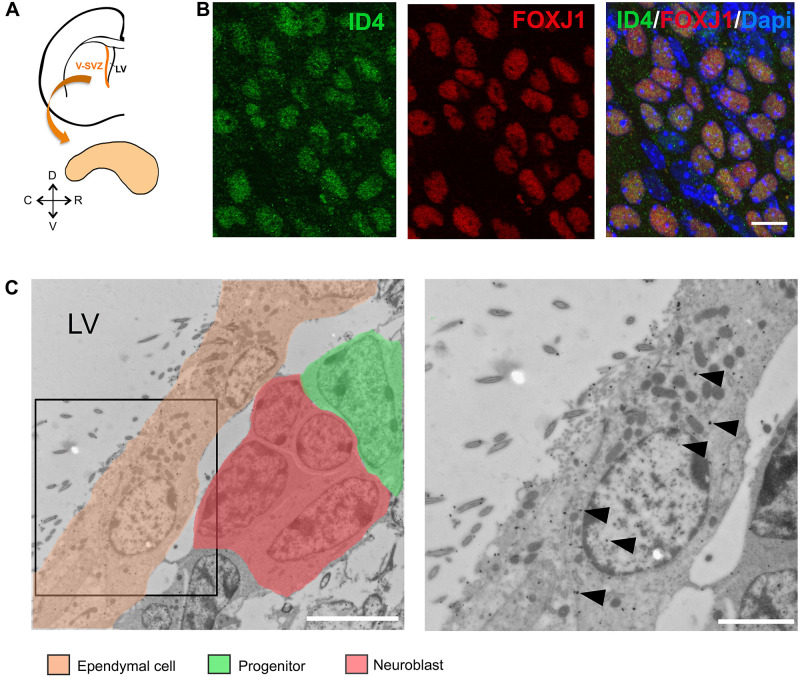
ID4 expression in ependymal cells. **(A)** Scheme of a coronal section of a mouse brain hemisphere showing the ventricular-subventricular zone (V-SVZ) of the lateral ventricles (LV) and a wholemount section that can be dissected out from this region. **(B)** Immunolabeling of ID4 and FOXJ1 (ependymal cell marker) on wholemount sections of adult C57BL/6J mice. Scale bar: 5 μm. **(C)** Immunogold staining of ID4 protein on V-SVZ sections. Left panel shows ependymal cells next to neural progenitors and neuroblasts (identified by pseudo-colors). Arrowheads indicate some of the gold particles labeling ID4 protein in ependymal cells. Right panel shows higher magnification of boxed area on left panel. Scale bar: 5 μm left and 2 μm right.

We performed ID4 immunolabeling on wholemount preparations of the LV of adult C57BL/6J mice (Figure 1A). We detected the expression of ID4 protein in ependymal cells as can be observed by co-localization with FOXJ1 protein (Figure 1B). To confirm this observation, we performed immunogold labeling for the ID4 protein in ultrathin sections from the V-SVZ. Several cell types, such as progenitor stem cells and neuroblasts were positive for ID4-immunogold (Figure 1C indicated by distinct pseudo-colors). In addition, ID4 labeling was detected in ependymal cells lining the LV confirming our immunofluorescence results (arrowheads in Figure 1C).

### *Id4−/−* Mice Display Defects in LV Development and Ependymal Cells Function

**FIGURE 2:**
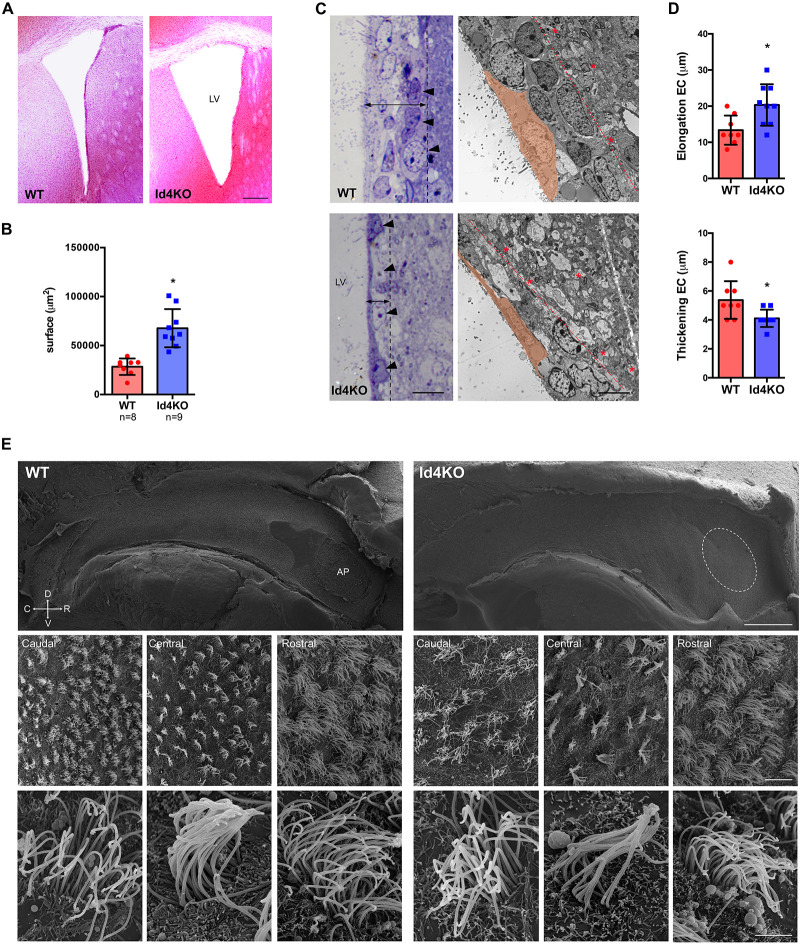
Absence of ID4 during development results in enlarged ventricles. **(A)** Hematoxylin-eosin staining of brain coronal sections from *Id4*+/+ (WT) and *Id4–/–* (Id4KO) adult mice showing enlarged lateral ventricles (LV). Scale bar: 200 μm. **(B)** Quantification of the surface of the LV in WT and Id4KO mice. *p-value ≤ 0.05. **(C)** Left, semithin sections of WT and Id4KO ventricular walls, stained with toluidine blue. Id4KO mice present a thinner subventricular wall delimited by dotted line, arrowheads indicate the outermost layer of cells in the V-SVZ. Right, transmission electron microscopy images shows elongation and thickening of ependymal cells and thinner LV delimited by dotted line and the presence of myelin sheets in Id4KO mice (asterisks). Scale bar: 5 μm. **(D)** The length and thickness of ependymal cells was measured on the LV of electron microscopy images of WT and Id4KO animals. *p-value ≤ 0.05. **(E)** Scanning electron microscopy of wholemount preparations from WT and Id4KO mice show enlargement of the ventricles and disappearance of the adhesion point (AP; circled), decreased ependymal cell density and altered organization in cilia. Scale bar from top to bottom: 500 μm, 5 μm and 2 μm.

In order to investigate the role of ID4 in ependymal cells, we analyzed the V-SVZ of *Id4−/−* mice (Id4KO) (Yun et al., 2004). Id4KO mice consistently displayed enlarged ventricles (Figures 2A,B). This was associated with thinning of the ventricular wall and stretching of the ependymal cells, as observed by light microscopy in toluidine blue-stained semithin sections (Figure 2C). By electron microscopy, the V-SVZ is characterized by a high cell density organized in four cell layers (Doetsch et al., 1997) (delimited by dotted line in Figure 2C, right panel) compared to the adjacent brain parenchyma displaying lower cell density and abundance of myelin sheets (asterisks in Figure 2C, right panels). Ependymal cells were more elongated and thinner in Id4KO animals, as shown by quantifications on electron microscopy images (Figure 2D). Scanning electron microscopy of wholemount preparations revealed the absence of adhesion point –the area where lateral and medial ventricle walls adhere to each other in *Id4−/−* mice (Figure 2E). In addition, we observed that ependymal cell density was decreased in all three rostral, central and caudal areas of the ventricle wall but we did not observe obvious differences in cilia shape or length.

**FIGURE 3:**
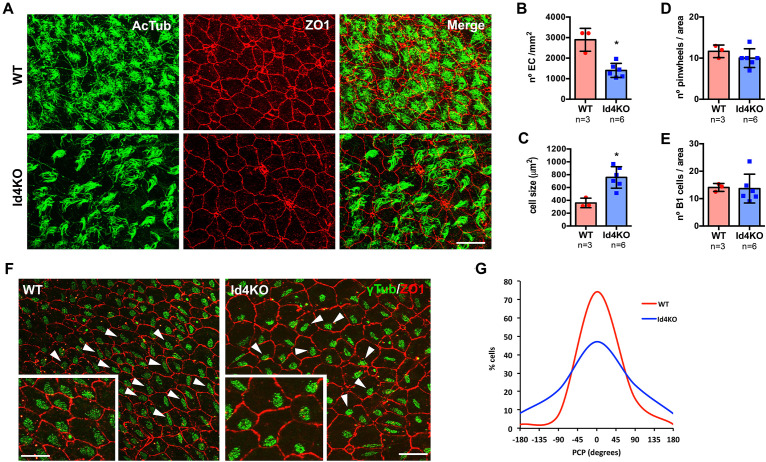
ID4KO mice present altered ependymal cells and disrupted planar cell polarity (PCP). **(A)** Immunofluorescence for ZO1 (red) and acetylated tubulin (green) in wholemount preparations of WT and Id4KO mice. Scale bar: 10 μm. **(B)** Quantification of the number of ependymal cells determined by ZO1 staining and **(C)** ependymal cell surface. **(D)** Quantification of the number of pinwheels and **(E)** the number of cells presenting one γ-tubulin staining inside pinwheels, identifying B1 astrocytes. **(F)** Immunofluorescence for ZO1 (red) and γ-tubulin (green) in wholemount preparations show disorganized PCP. Arrowheads indicate the orientation of the BB to respect the cell wall. Scale bar: 10 μm, 5 μm for insets. **(G)** Measurement of the PCP in the wholemount preparations of WT and Id4KO mice. *p-value ≤ 0.05.

To confirm a decrease in ependymal cell density, we performed immunofluorescent staining on wholemount preparations of the ventricle wall (labeling tight junctions with ZO-1 antibody) and cilia (acetylated tubulin, 6-11B) in WT and Id4KO mice (Figure 3A). The density of ependymal cells was significantly decreased in Id4KO mice together with an increase in the cell surface when compared the same regions (Figures 3B,C). Despite higher intensity of ZO-1 staining, we found no notable differences in the number of pinwheels or the number of monociliated cells (corresponding to B1 cells) in the center of the pinwheels (Figures 3D,E). Several reports have linked PCP disruption with CSF flow alterations (Mirzadeh et al., 2010; Jiang et al., 2020). To investigate whether PCP of ependymal cells could be altered in Id4KO brains, we measured cilia BB orientation (Mirzadeh et al., 2010). Basal bodies were labeled with anti-γ-tubulin (GTU88) antibody and their orientation was determined relative to the ependymal cell wall labeled with anti-ZO1 antibody. We noticed that the organization of BB patches was altered in the Id4KO mice (indicated by arrowheads in Figure 3F), with a significant decrease of the median orientation (Figure 3G).

### Id4 Deletion During Embryogenesis Impacts on Ependymal Cell Development

**FIGURE 4:**
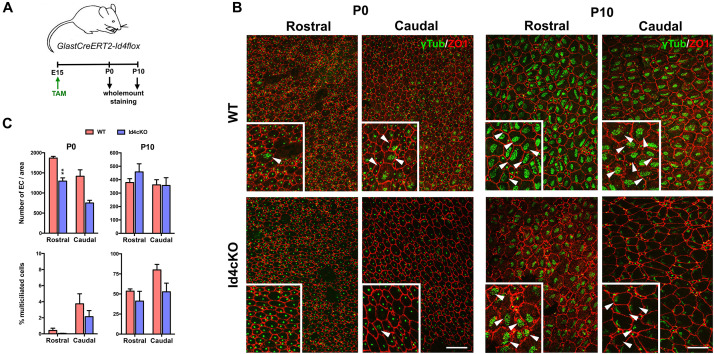
Absence of ID4 results in delayed ependymal cell maturation. **(A)** Scheme of the experimental set-up. **(B)** Wholemount staining of P0 and P10 mice for Z01 (red) and γ-tubulin (green) showing defective ependymal cell development in rostral and caudal regions in Id4cKO. Mature multiciliated cells are indicated by arrowheads. Scale bar: 10 μm, 15 μm for insets. **(C)** Quantifications at P0 and P10 of the number of ependymal cells and the fraction of mature ependymal cells (% of multiciliated cells) at the time of the analysis in rostral and caudal regions of the wholemount preparations. **p-value ≤ 0.01.

Ependymal cells differentiate from radial glia cells at E14-16 and maturation occurs from caudal to rostral orientation during the first postnatal weeks (Spassky et al., 2005), during which the primary cilium is replaced by multiple motile cilia (9 + 2 microtubule doublets) (Mirzadeh et al., 2010). To evaluate whether ID4 was involved in ependymal cell differentiation and/or maturation we induced ID4 deletion in *GlastCreERT2-Id4flox (Id4cKO)* mice at E15 and analyzed their phenotype at P0 to evaluate differentiation and at P10 to analyze maturation (Figure 4A). We performed immunofluorescence for γ-tubulin (GTU88) to identify the BB and for Z01 to identify the cell wall in wholemount preparations at both P0 and P10 in rostral and caudal regions (Figure 4B). At P0, the number of ependymal cells was significantly decreased in the Id4cKO notably in the rostral area (Figure 4C), suggesting that ID4 may be involved in differentiation of ependymal cells from radial glial cells at late embryonic stages. We next evaluated ependymal cell maturation by measuring the proportion of ependymal cells with multiple motile cilia. The presence of multiciliated cells is very low at P0 when maturation starts. In contrast, at P10, ependymal cells display a characteristic BB pattern indicative of maturation (Spassky et al., 2005). We observe decreased fraction of multiciliated cells in rostral regions of the ventricle wall in Id4cKO compared to control animals. Together, our data suggest that ID4 may be important for ependymal cell maturation and correct cilia development.

## Discussion

ID4 plays an essential role in correct neural cell differentiation and maturation during embryogenesis (Yun et al., 2004; Bedford et al., 2005). In this work, we present evidence that ID4 might also be important for development and maturation of ependymal cells from radial glial cells. First, we describe for the first time that ID4 protein is expressed in ependymal cells. Absence of ID4, initiated at early stages of brain development, resulted in altered ependymal cell layer cytoarchitecture, decreased ependymal cell numbers and enlarged ventricles as a consequence. The absence of adhesion point was also a constant in Id4KO mice and was already reported to be linked to hydrocephalus in other mutants, such as KIF3A-deficient mice (Mirzadeh et al., 2010). Early inactivation of ID4 at E15 during differentiation of ependymal cells from radial glial cells resulted in decreased number of ependymal cells at P0. The adult Id4KO presented a similar defect in ependymal cell numbers, suggesting that ID4 may be important in ependymal cell determination from radial glial cells. Defects in differentiation from radial glial cells were also observed when the forkhead transcription factor FOXJ1 was absent (Jacquet et al., 2009). GemC1 – a regulator of the DNA replication– is another factor playing an important role in the ependymal cell-neural stem cell balance (Ortiz-Álvarez et al., 2019).

In addition to a defective differentiation, our findings suggest that ID4 may also be important for correct timing on ependymal cell maturation. Deletion of *ID4* at E15 resulted in a decreased fraction of mature multiciliated ependymal cells (presenting multiple BB) at P10, suggesting delayed maturation compared to control animals. We also observed that the orientation of basal body patches was different in the absence of ID4, suggesting an altered PCP. However, how these defects impact cilia function remains to be assessed in the context of ID4 absence. Decreased number and delayed maturation of ependymal cell beating capacity could lead to disrupted CSF flow dynamics early during brain development and accumulation of CSF. Accumulation of CSF within the brain ventricles due to defect in ependymal cell development is one of the mechanisms responsible of hydrocephalus (Ibañez-Tallon et al., 2004). Despite complete cilia development in ependymal cells, PCP seemed to be affected by the absence of ID4. This could suggest a potential role of ID4 in centrosome organization or cilia motility. Malfunction in ependymal cell beating activity can impair CSF clearance and cause excessive accumulation within the ventricles leading to hydrocephalus as a result of increased pressure. Besides, CSF flow dynamics can also impact NSC proliferation and neuroblast migration toward the OB. Several studies have reported that the NSC’s primary cilium works as an “antenna” sensing the changes in CSF flow (Silva-Vargas et al., 2016). CSF flow generates protein gradients contributing with vector information for migratory neuroblasts (Sawamoto et al., 2006). Therefore, disruption of CSF caused by genetic defects may also indirectly impact NSC homeostasis and/or migration.

Our findings here show for the first time a role of ID4 in ependymal cell differentiation and maturation. Further investigation should be conducted to better understand the mechanism leading to such phenotype, the potential protein partners of ID4 and the impact on neurogenesis and neuroblast migration.

## Data Availability Statement

The raw data supporting the conclusions of this article will be made available by the authors, without undue reservation.

## Ethics Statement

The animal study was reviewed and approved by the French Ministry of Research and Higher Education 3572-201601 0817294743 v5.

## Author Contributions

BR and EH: conceptualization, methodology, and writing – original draft. BR, and VH-P: investigation. JG-V, VH-P, BR, and EH: funding acquisition. JG-V and EH: supervision. All authors: writing – review and editing.

## Conflict of Interest

The authors declare that the research was conducted in the absence of any commercial or financial relationships that could be construed as a potential conflict of interest.
